# ﻿Three new species of *Cortinarius* section *Delibuti* (Cortinariaceae, Agaricales) from China

**DOI:** 10.3897/mycokeys.101.114705

**Published:** 2024-01-17

**Authors:** Pan Long, Song-Yan Zhou, Sai-Nan Li, Fei-Fei Liu, Zuo-Hong Chen

**Affiliations:** 1 College of Life Science, Hunan Normal University, Changsha 410081, China Hunan Normal University Changsha China; 2 Key Laboratory for Plant Diversity and Biogeography of East Asia, Kunming Institute of Botany, Chinese Academy of Sciences, Kunming 650201, China Kunming Institute of Botany, Chinese Academy of Sciences Kunming China

**Keywords:** Morphology, new taxa, phylogeny, taxonomy

## Abstract

Three new species of CortinariussectionDelibuti, namely *C.fibrillososalor*, *C.pseudosalor*, and *C.subtropicus* are described as new to science based on morphological and phylogenetic evidences. *Cortinariuspseudosalor* is extremely morphologically similar to *C.salor*, but it differs from the latter by smaller coarsely verrucose basidiospores. *Cortinariusfibrillososalor* can be easily differentiated by its fibrillose pileus. The pileus of *C.subtropicus* becomes brown without lilac tint at maturity comparing with other members of section Delibuti. A combined dataset of ITS and LSU sequences was used for phylogenetic analysis. The phylogenetic reconstruction of section Delibuti revealed that these three new species clustered and formed independent lineages with full support respectively. A key to the three new species and related species of section Delibuti is provided in this work.

## ﻿Introduction

The genus *Cortinarius* (Pers.) Gray (Cortinariaceae, Agaricales), which is known for its high species diversity, comprises more than 3000 taxa and exhibits a global distribution (Garnica et al. 2005; Willis 2018). However, the taxonomy of this genus faces an extremely complex challenge due to the overlapping morphological variation within species (Seidl 2000; Dima et al. 2021). Different classification systems of *Cortinarius* have been proposed by many taxonomists based on the comparison of the morphological characteristics, geographical distribution, ecological traits, chemical features, DNA barcode markers, or diverse combinations of the above through introducing the infrageneric concepts such as subgenus, section, or clade (Moser 1969; Moser and Horak 1975; Singer 1986; Bidaud et al. 1994; Brandrud 1998; Peintner et al. 2002; Garnica et al. 2003; Peintner et al. 2004; Garnica et al. 2005; Stefani et al. 2014; Garnica et al. 2016; Niskanen et al. 2016; Soop et al. 2019). Recently, according to the data of shallow whole genome sequencing and a five-locus analysis of 245 species, the genus *Cortinarius* was elevated to the Cortinariaceae rank, encompassing 10 genera, namely *Aureonarius* Niskanen & Liimat., *Austrocortinarius* Niskanen & Liimat., *Calonarius* Niskanen & Liimat., *Cortinarius*, *Cystinarius* Niskanen & Liimat., *Hygronarius* Niskanen & Liimat., *Mystinarius* Niskanen & Liimat., *Phlegmacium* (Fr.) Wünsche, *Thaxterogaster* Singer and *Volvanarius* Niskanen & Liimat. (Liimatainen et al. 2022).

Cortinariussect.Delibuti (Fr.) Sacc., typified by *C.delibutus* Fr., is widely distributed (Høiland and Holst-Jensen 2000; Peintner et al. 2004). section Delibuti species possess a viscid to glutinous pileus and glutinous cylindrical to clavate stipe, a duplex pileipellis with a gelatinous layer, subglobose and moderately warty basidiospores, basidiome in shades of bluish, yellow, brown, or green, lilac-blue lamellae while brown in the mature stage and a ring zone usually on the upper part of the stipe (Peintner et al. 2004; Garnica et al. 2005; Soop et al. 2019). As a comparatively old lineage, sect. Delibuti used to be placed in the myxacioid group or subgenus Myxacium (Fr.) Trog (Singer 1986; Brandrud et al. 1990, 1992; Seidl 2000; Garnica et al. 2005). In other views, sect. Delibuti was also placed in phlegmacioid group or subg. Phlegmacium, including subsections *Delibuti* and *Anomali* (Antonio and Aguirre 2004; Peintner et al. 2004). Based on four- locus (nrITS, nrLSU, *rpb1*, and *rpb2*) phylogenetic analysis, sect. Delibuti was placed within larger entity-Anomaloid sections, including sections *Anomali*, *Bolares*, *Delibuti*, *Spilomei* and *Subtorti* (Soop et al. 2019). More recently, sect. Delibuti was placed in Cortinariussubgen.Camphorati Liimat., Niskanen & Ammirati, encompassing sections *Anomali*, *Bolares*, *Lilacinocinerei* and *Subtorti* by Liimatainen et al. (2022).

The research on *Cortinarius* has mainly been conducted in Europe and North America, while it is still lacking in East Asia (Peintner et al. 2002; Garnica et al. 2003; Peintner et al. 2004; Garnica et al. 2005; Liimatainen et al. 2014; Stefani et al. 2014; Garnica et al. 2016; Niskanen et al. 2016; Soop et al. 2019, Liimatainen et al. 2022). To date, fewer than 30 species were originally reported from China, and only two new species in sect. Delibuti were originally found in China (Yang 1998; Wei and Yao 2013; Xie et al. 2019, 2020, 2021a, 2021b, 2022; Luo and Bau 2021, Zhang et al. 2023; Zhou et al. 2023). With the combination of morphological observations and phylogenetic analysis, we describe three species belonging to sect. Delibuti as new to science in this study.

## ﻿Materials and methods

### ﻿Specimens

The specimens were collected from central and southwestern China during 2012–2022. The vouchers are all deposited in the Mycological Herbarium of Hunan Normal University (MHHNU) and Cryptogamic Herbarium of Kunming Institute of Botany, Chinese Academy of Sciences (KUN-HKAS). Detailed information is listed in Table 1.

**Table 1.** List of sequences of *Cortinarius* used for phylogenetic analyses. The sequences newly generated in this study are in bold, and all type specimens are highlighted with an asterisk.

Species	Voucher	Locality	GenBank Accession No.		Reference
			ITS	LSU	
*Cortinariusanomalus*	TUB011883	Europe, Germany	AY669645	AY669645	Garnica et al. (2005)
C.anomalus*	CFP1154 (S)	Europe, Ångermanland	KX302224	–	Dima et al. (2016)
C.barlowensis*	JFA13140	North America	FJ717554	–	Harrower et al. (2011)
*C.bolaris*	T40	Europe, Norway	KC842426	KC842496	Stensrud et al. (2014)
C.bolaris*	CFP1008	Europe	KX302233	–	Dima et al. (2016)
*C.bolaris*	TUB0118524	Europe, Germany	AY669596	AY669596	Garnica et al. (2005)
C.calaisopus*	PDD 94050	New Zealand, Dunedin	NR157880	MH108373	Genbank
*C.calaisopus*	PDD103678/CO2106	New Zealand	KF727395	KF727338	Soop et al. (2019)
*C.camphoratus*	SMI193	North America, Canada	FJ039626	–	Harrower et al. (2011)
*C.delibutus*	F17048	North America, Canada	FJ717515	FJ717515	Harrower et al. (2011)
*C.delibutus*	SAT01-301-12	North America, USA	FJ717513	–	Harrower et al. (2011)
*C.dysodes*	PDD70499/CO1038 HT	New Zealand	GU233340	GU233394	Soop et al. (2019)
C.ferrusinus*	JB8106 13	Europe	KY657254	–	Genbank
***C.fibrillososalor*** *	**MHHNU 32494**	**East Asia, China, Hunan**	**OR647481**	**OR647506**	**This study**
***C.fibrillososalor***	**MHHNU 33520**	**East Asia, China: Hunan**	**OR647485**	**OR647507**	**This study**
***C.fibrillososalor***	**MHHNU 33509**	**East Asia, China, Hunan**	**OR647483**	–	**This study**
***C.fibrillososalor***	**MHHNU 8657**	**East Asia, China, Hunan**	**OR647355**	**OR647497**	**This study**
***C.fibrillososalor***	**MHHNU 32070**	**East Asia, China, Hunan**	**OR660685**	**OR647503**	**This study**
*C.illibatus*	HMJAU48760	East Asia, China, Heilongjiang	MW911735	–	Xie et al. (2021a)
*C.illibatus*	OS574	Europe	KC842441	KC842511	Stensrud et al. (2014)
C.pseudocamphoratus*	HMJAU48694	East Asia, China, Xizang	NR_176776	–	Xie et al. (2022)
*C.putorius*	TN07411 HT	North America, USA	KR011124	–	Ariyawansa et al. (2015)
*C.rotundisporus*	PDD96298/ JAC12057	New Zealand	MH101550	MH108389	Soop et al. (2019)
*C.rotundisporus*	PERTH 05255074	Australia	AY669612	AY669612	Garnica et al. (2005)
*C.salor*	TUB011838	Europe, Germany	AY669592	AY669592	Garnica et al. (2005)
C.spilomeus*	S: CFP1137	Europe	KX302267	–	Dima et al. (2016)
*C.spilomeus*	TUB011523	Europe	AY669654	AY669654	Garnica et al. (2005)
***C.pseudosalor***	**MHHNU 8349**	**East Asia, China, Hunan**	**OR647352**	–	**This study**
***C.pseudosalor*** *	**MHHNU 32082**	**East Asia, China, Hubiei**	**OR660686**	**OR647504**	**This study**
***C.pseudosalor***	**MHHNU 32148**	**East Asia, China, Hubiei**	**OR660688**	**OR647505**	**This study**
*C.subsalor*	HMJAU48758	East Asia, China, Zhejiang	MW911733	–	Xie et al. (2021a)
C.subsalor*	HMJAU48759	East Asia, China, Zhejiang	MW911734	–	Xie et al. (2021a)
*C.subtortus*	F16111	North America	FJ157044	FJ157044	Harrower et al. (2011)
*C.subtortus*	TUB011382	Europe	AY174857	AY174857	Garnica et al. (2003)
***C.subtropicus***	**MHHNU 31954**	**East Asia, China, Hunan**	**OR647356**	**OR647498**	**This study**
***C.subtropicus***	**KUN-HKAS 75760**	**East Asia, China, Guangxi**	**OR647491**	**OR647509**	**This study**
***C.subtropicus***	**MHHNU 31964**	**East Asia, China, Hunan**	**OR660684**	**OR647501**	**This study**
***C.subtropicus***	**MHHNU 31981**	**East Asia, China, Hunan**	**OR660687**	**OR647502**	**This study**
***C.subtropicus*** *	**MHHNU 33533**	**East Asia, China, Hunan**	**OR647488**	**OR647508**	**This study**
*C.tasmacamphoratus*	HO A20606A0	Australia, Tasmania	AY669633	AY669633	Garnica et al. (2005)
*C.tessiae*	PDD107517/CO1450	New Zealand	MG019356	MG019356	Soop et al. (2019)
*C.tessiae*	PDD72611	New Zealand	HM060317	HM060316	Genbank
C.tetonensis*	JFA10350	North America	MZ580436	–	Dima et al. (2016)
C.tibeticisalor*	HMJAU48764	East Asia, China, Xizang	MW911730	–	Xie et al. (2021a)
*C.tibeticisalor*	HMJAU48762	East Asia, China: Xizang	MW911731	–	Xie et al. (2021a)
*C.tibeticisalor*	HMJAU48763	East Asia, China, Xizang	MW911732	–	Xie et al. (2021a)
*C.viridipileatus*	OTA61977	New Zealand	MK546592	MK546595	Nilsen et al. (2021)
*C.viridipileatus*	OTA64087	New Zealand	MK546593	MK546596	Nilsen et al. (2021)

### ﻿Morphological observation

The descriptions of macromorphological characters were based on field records and photographs. Color codes were used following Kornerup and Wanscher (1978). The size of basidiomes, as determined by pileus width, was described as small (< 5.0 cm), medium-sized (5.0–9.0 cm) or large (> 9.0 cm). Microscopic features were observed from dried specimens that were mounted with 5% aqueous KOH and stained with 1% Congo red solution under a light microscope (Motic Ltd., China). Melzer’s reagent was used as an indicator of the amyloidity of basidiospores. In the description of basidiospores, the abbreviation [n/m/p] represents that the measurements were made on n basidiospores from m basidiomes of p collections. At least twenty matured basidiospores and basidia from each of the basidiomes were measured. The range (a)b–c(d) stands for the dimensions of basidiospores in which b–c contains a minimum of 90% of the measured values, while a and d indicate the extreme values. In addition, a Q value shows the ratio of length to width of basidiospores, and a Qm value shows the average Q ± standard deviation. A JSM-6380LV scanning electron microscope (JEOL Ltd., Tokyo, Japan) was used for the observation of ornamentations of basidiospores.

### ﻿DNA extraction, PCR amplification and sequencing

Total genomic DNA was extracted by a Fungal DNA Mini Kit (Omega, USA). ITS 4 and ITS 5 (White et al. 1990), LROR and LR5/LR7 (Vilgalys and Hester 1990), were used for amplification of internal transcribed spacer (ITS), nuclear ribosomal large subunit (nrLSU), respectively. Each PCR mixture contained 1× PCR buffer, 1.5 mM MgCl2, 0.2 mM dNTPs, 0.4 μM of each primer, 1.25 U of Taq polymerase, and 1–2 μl DNA template in a total volume of 25 μl. PCRs were performed with an Eppendorf Mastercycler thermal cycler (Eppendorf Inc., Germany) as follows: initial denaturation at 94 °C for 4 min (ITS; nrLSU; *tef1-α*); followed by 30–35 cycles of 94 °C for 30 s (ITS; nrLSU), 54 °C for 30 s (ITS) or 55 °C for 1 min (nrLSU), and 72 °C for 30 s (ITS), 1 min (nrLSU); and a final extension at 72 °C for 7–10 min. Amplified PCR products were detected by gel electrophoresis on 2% agarose gels and then sent to Tsingke Biological Technology (China) for sequencing.

### ﻿Phylogenetic analyses

The sequences newly generated in this study and downloaded from GenBank were used for phylogenetic analysis (Table 1). Alignment was performed by MAFFT v7.149b (Katoh and Standley 2013) and adjusted manually by MEGA5 (Tamura et al. 2011). SequenceMatrix 1.7.8 (Vaidya et al. 2011) was applied to generate multigene matrixes. GTR+I+G was selected as the best-fit model for combined matrix based on the Akaike Information Criterion (AIC) by MrModeltest 2.3 (Nylander 2004). Maximum likelihood (ML) analysis was performed using the W-IQ-TREE web service (http://iqtree.cibiv.univie.ac.at/) with 1000 ultrafast bootstrap replicates (Trifinopoulos et al. 2016). Bayesian inference (BI) was performed in MrBayes v3.2 (Ronquist et al. 2012). Four Metropolis-coupled Monte Carlo Markov chains were run for 5000000 generations, sampling every 1000^th^ generation. Subsequently, the sampled trees were summarized after omitting the first 25% of trees as burn-in.

## ﻿Results

### ﻿Phylogenetic analyses

**Figure 1.**
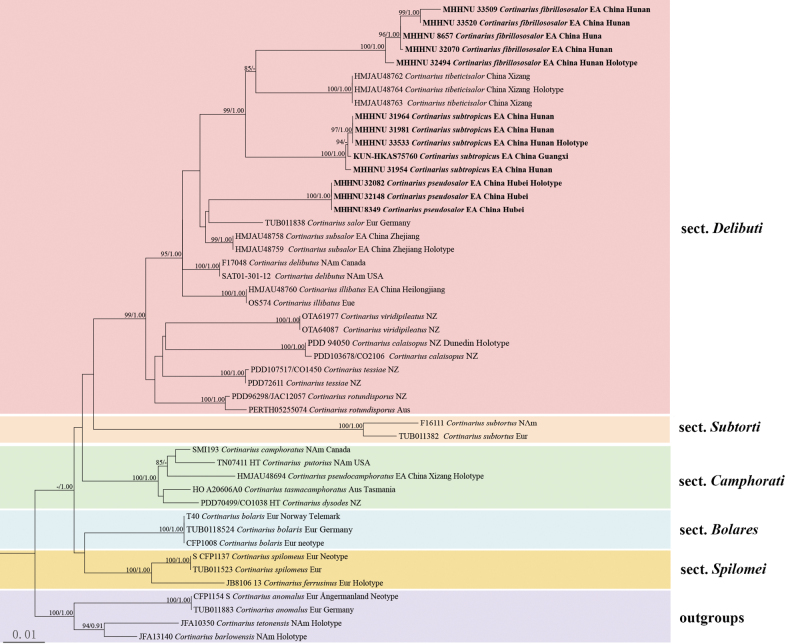
Phylogenetic tree of Cortinariussect.Delibuti inferred from a combined matrix of ITS and LSU through maximum likelihood and Bayesian inference. Bayesian posterior probabilities (PP) > 0.90 and bootstrap values (BP) >85% are reported at the nodes (PP/BP); “–” indicates that the support value was less than the respective threshold. The three newly described species are highlighted in bold. Aus: Australia; EA: East Asia; Eur: Europe; NAm: North America; NZ: New Zealand.

In the concatenated dataset (ITS+LSU), a total of 78 sequences (48 ITS, 30 LSU) from 48 samples were used for phylogenetic analyses among sect. Delibuti, sect. Subtorti, sect. Camphorati, sect. Bolares, sect. Spilomei, and sect. Anomali, of which 24 sequences (13 ITS, 11 LSU) were newly yielded in this study (Table 1). The estimates of tree topology inferred from ML and Bayesian analyses were extremely similar. The ML phylogenetic tree is shown with both bootstrap values (BP) and posterior probabilities (PP) annotated near the nodes (Fig. 1).

The phylogenetic relationship of sections within the genus *Cortinarius* in the present study was unclear and weakly supported. In the multi-locus tree, the monophyly of sect. Delibuti was supported with well-supported values (BP = 99%, PP = 1.00), including 12 species. section Camphorati was also monophyletic with fully supported values (BP = 100%, PP = 1.00), emcompassing 5 species. In sect. Delibuti, *C.delibutus*, *C.illibatus*, *C.salor*, *C.subsalor*, *C.tibeticisalor*, and three novel species, namely *C.fibrillososalor*, *C.pseudosalor*, and *C.subtropicus*, formed a monophyletic lineage (BP = 95%, PP = 1.00). *Cortinariusfibrillososalor*, *C.subtropicus*, and *C.tibeticisalor* formed a clade only be found in East Asia (BP = 99%, PP = 1.00), while *C.tibeticisalor* has a special olive-green tint, was only distributed in Tibetan Plateau (Xie et al. 2021a). However, *C.pseudosalor*, *C.salor* and *C.subsalor* clustered with low support values, leaving the position not determined. In addition, 13 specimens from *C.pseudosalor*, *C.fibrillososalor* and *C.subtropicus* collected in this study were fully supported (BP = 100%, PP = 1.00), and the phylogenetic relationships of *C.fibrillososalor*, *C.tibeticisalor* and *C.subtropicus* were clarified (BP = 100%, PP = 1.00).

### ﻿Taxonomy

#### ﻿ Cortinarius fibrillososalor

Taxon classificationFungiAgaricalesCortinariaceae

P. Long & Z.H. Chen sp. nov.

BB04344F-3F7F-5092-BE9B-7E02A8DADC6E

850393

Figs 2 , 3

##### Etymology.

*Fibrillososalor* (Latin) refers to the species morphologically similar to *Cortinariussalor*, but with fibrils on the pileus.

##### Holotype.

China, Hunan Province: Sangzhi County, Badagongshan National Nature Reserve, at 29.782541°N, 110.084472°E, alt. 1424 m, 8 September 2020, Z.H. Chen, P. Long and S.N. Li, (MHHNU 32494).

**Figure 2.**
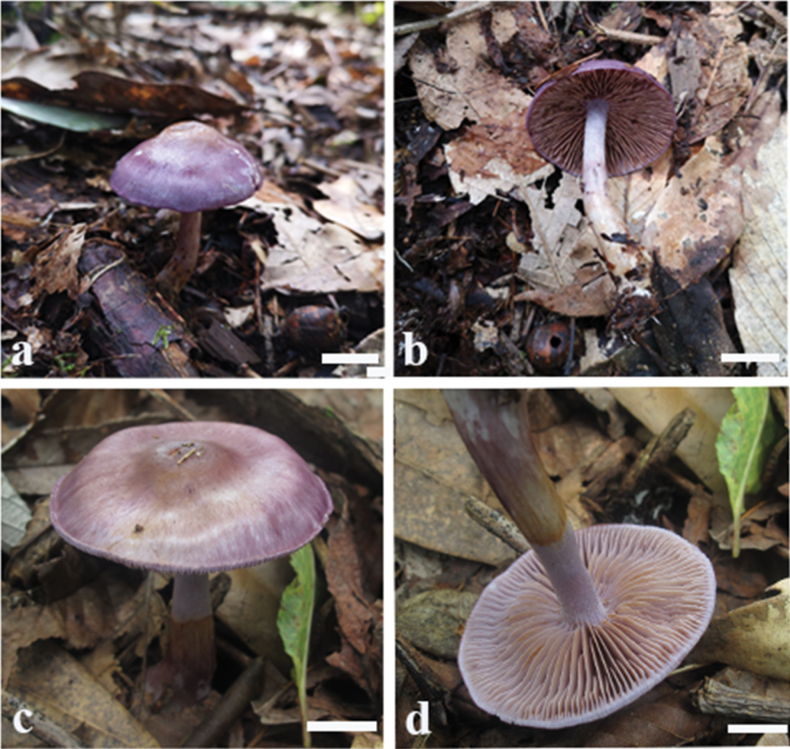
Basidiomes of *Cortinariusfibrillososalor* (**a, b**MHHNU 32494 **c, d**MHHNU 8657).

##### Diagnosis.

Differs from the other species of sect. Delibuti from its fibrillose pileus.

**Figure 3.**
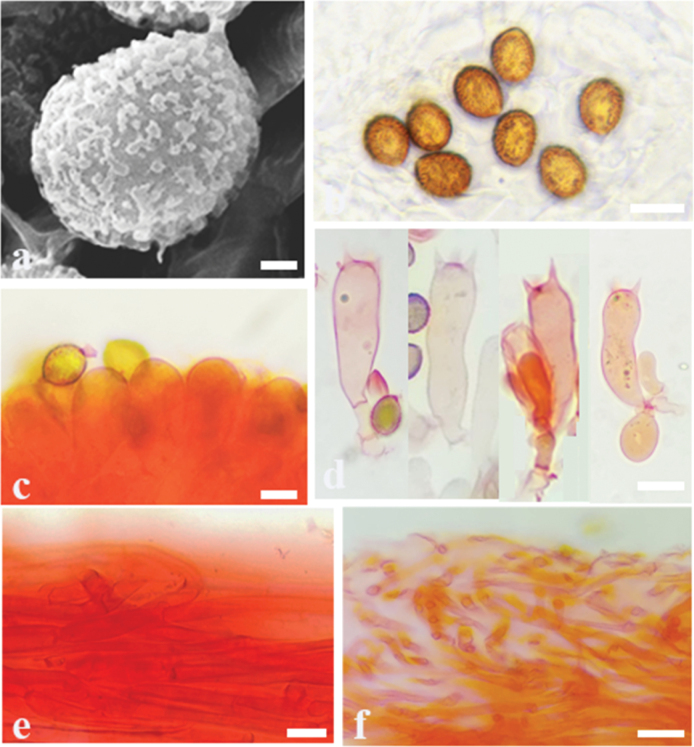
Microscopic features of *Cortinariusfibrillososalor* (MHHNU 32494) **a** scanning electron micrograph of basidiospore **b** basidiospores **c** lamellae edge **d** basidia with probasidium **e** stipitipellis **f** pileipellis; Scale bars: 1 μm (**a**); 10 μm (**b–e**); 20 μm (**f**).

##### Description.

Basidiomes small to medium-sized, telamonia-like, development type stipiocarpic. Pileus 2.9–5.2 cm, at first broadly convex, then lower convex to plane, broadly umbonate at the centre, margin incurved or decurved to upturned; at first violaceous (17B6–17B8), tinged brown (5B4–5C6) at the centre then becoming whitish mauve (16A1–16A2), finely fibrillose, with brown (5A5–5C7) universal veil remains at margin; surface silky when dry or glutinous when wet. Context thin, creamy white, soft, beige (3A1–A2) when bruised. Lamellae adnate to adnexed, lilac (17A2–17B2) to brownish (6C5–6D7), moderately distant, sometimes margin wavy. Stipe cylindrical to clavate, bend, gradually slender to the apex, 3.4–5.9 cm long, 0.4–0.8 cm wide, violaceous (17A4–17B5) when young then fading to whitish mauve (16A2–16A3) tint, leaving an ochraceous (5B6–5D8) ringon the upper stem, hollow. Odour indistinct.

Basidiospores [100/5/5] (6.5–) 7.0–8.8 (–9.2) × (5.0–) 5.9–7.2 (–8.1) μm, av. 8.1 × 6.5 μm, Q = 1.14 (1.16) – 1.31 (1.45), Qm = 1.24 ± 0.02, broadly globose to long ellipsoid, rarely subglobose, yellowish brown, moderately verrucose, without amyloid and dextrinoid reaction. Basidia (27–) 28–35 × (8–) 9–11 μm, 4-spored, sterigmata up to 2.4–3.7 μm, clavate to subcylindrical, colourless or with amber yellow oily inclusions or granules. Pileipellis duplex, hyphae 4–8 μm wide, epicutis strongly gelatinous, 68–128 μm thick, composed of colourless or amber yellow, irregularly arranged and strongly interwoven hyphae, hypocuits 25–38 μm thick, composed of colourless or amber yellow, nearly parallel cylindrical hyphae. Lamellar edges fertile. Cystidia absent. Lamellar trama regular, 40–80 μm thick, composed of parallel arranged hyphae, hyphae 3–6 μm wide. Stipitipellis gelatinous, stipe hyphae 3–6 μm wide, thin-walled, cylindrical, interwoven. Clamp connections present in all tissues.

##### Habitat, ecology and distribution.

Solitary to gregarious on soil in evergreen broad-leaved forest, known from Hunan, China; July to September.

##### Additional specimens examined.

China, Hunan Province: Sangzhi County, Badagongshan National Nature Reserve, at 29.769154°N, 110.086577°E, alt. 1405 m, 31 July 2020, Z.H. Chen, P. Long and S.N. Li, (MHHNU 32070). China, Hunan Province: Sangzhi County, Badagongshan National Nature Reserve, at 29.769154°N, 110.477086°E, alt.1482 m, 28 July 2022, Z.H. Chen, J. Wen and Z.J. Jiang (MHHNU 33509, MHHNU 33520). China, Hunan Province: Sangzhi County, Badagongshan National Nature Reserve, at 29.404913°N, 109.491158°E, alt. 1500 m, 10 September 2015, P. Zhang, (MHHNU 8657).

##### Notes.

*Cortinariusfibrillososalor* can be differentiated from other species of section Delibuti for its fibrillose pileus, usually under evergreen broad-leaved forest at 1405–1500m. In addition, basidiospores broadly globose to long ellipsoid, rarely subglobose while other members in this section usually subglobose to broadly ellipsoid.

#### ﻿ Cortinarius pseudosalor

Taxon classificationFungiAgaricalesCortinariaceae

P. Long & Z.H. Chen sp. nov.

946C3CE9-E812-5383-85AD-BF278962EE20

 850392

Figs 4 , 5

##### Etymology.

*Pseudosalor* (Latin) refers to the species morphologically similar to *Cortinariussalor*.

##### Holotype.

China, Hubei Province: Hefeng County, Mulinzi National Nature Reserve, at 30.058935°N, 110.209541°E, alt.1413 m, 1 August 2020, Z.H. Chen, P. Long and S.N. Li, (MHHNU 32082).

**Figure 4.**
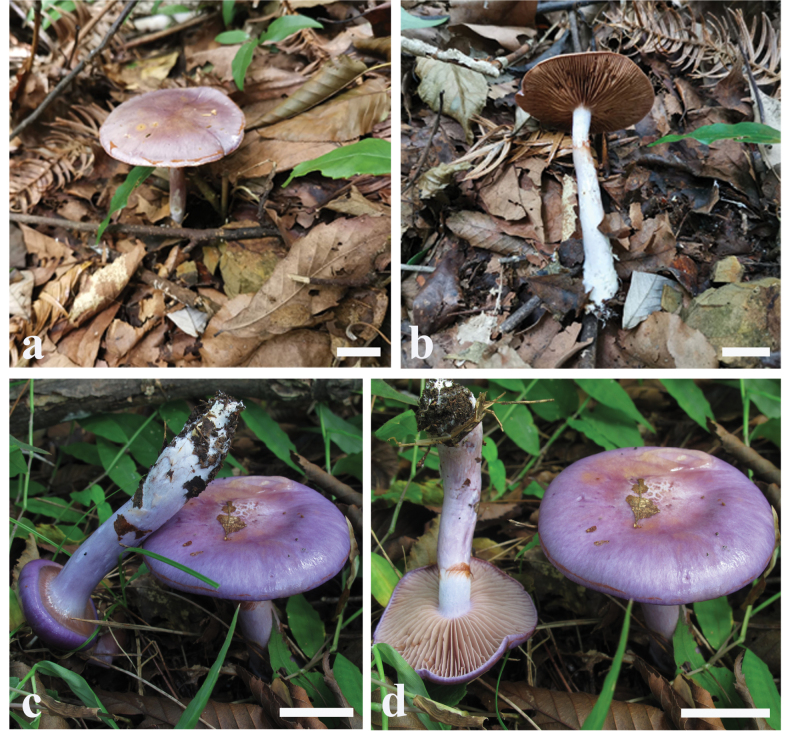
Basidiomes of *Cortinariuspseudosalor* (**a, b**MHHNU 32082 **c, d**MHHNU 8349).

##### Diagnosis.

This species differs from other species in sect. Delibuti for its high morphological similarity with *C.salor*, but having smaller coarsely verrucose basidiospores.

**Figure 5.**
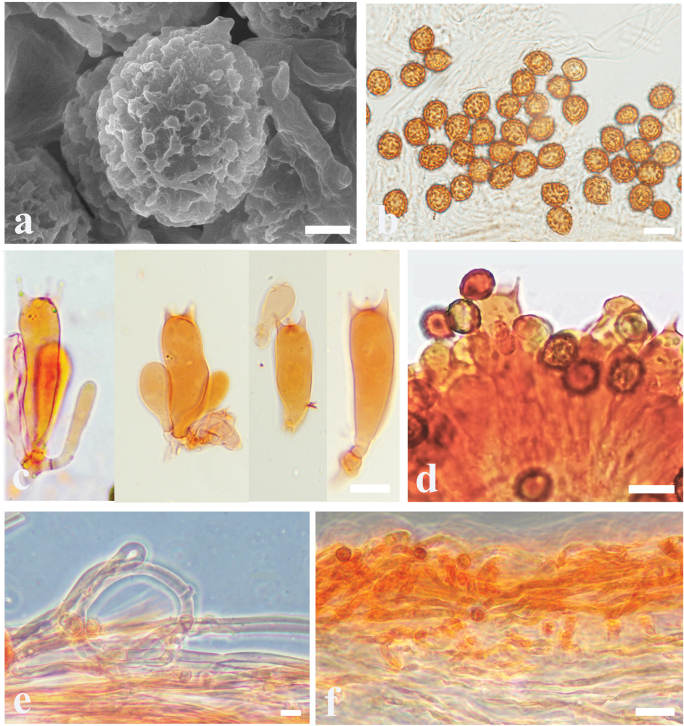
Microscopic features of *Cortinariuspseudosalor* (MHHNU 32082) **a** scanning electron micrograph of basidiospore **b** basidiospores **c** basidia with probasidium **d** lamellae edge **e** stipitipellis **f** pileipellis. Scale bars: 1 μm (**a**); 10 μm (**b–e**); 20 μm (**f**).

##### Description.

Basidiomes small to medium-sized, development type stipiocarpic. Pileus 2.8–6.5 cm, at first broadly convex, then lower convex to plane, margin incurved when young, decurved to upturned at maturity; bluish violaceous (18A3–18C5) when young, tinge of white at the centre when chapped, later fading to ochraceous grey (5B6–5C7) when old with brown (5B8–5C8) universal veil remains at margin; dry, viscid. Context dirty white, soft. Lamellae adnexed, pale yellow (1A2) with lilac tint (16A1–16A2) then brownish (5B6–5D7), moderately distant, sometimes margin wavy. Stipe clavate, gradually slender to the apex, 4–8.4 cm long, 0.4–1.0 cm wide, violaceous (16A2–16A4) when young then fading to upper dirty white, whitish mauve (16A2) at base, leaving an ochraceous ring (5B8–5C8) on the upper stem, hollow in centre. Odour indistinct.

Basidiospores [60/3/3] (7.3–) 7.4–8.4 × (5.7–) 6.0–7.4 (–7.5) μm, av. 7.9 × 6.7 μm, Q = (1.11) 1.12– (1.26) 1.27, Qm = 1.18 ± 0.11, subglobose to broadly ellipsoid, yellowish brown, coarsely verrucose, without amyloid and dextrinoid reaction. Basidia (29–) 30–38 × (8–) 9–12 μm, 4-spored, sterigmata up to 3.7–5.0 μm, clavate to subcylindrical, colourless or with amber yellow granules. Pileipellis duplex obviously, hyphae 2–6 μm wide, epicutis gelatinous, 50–75 μm thick, composed of colourless or amber yellow, moderately interwoven hyphae, hypocuits 50–75 μm thick, composed of colourless or amber yellow, hyphae nearly parallel cylindrical. Lamellar edges fertile. Cystidia absent. Lamellar trama regular, 45–55 μm thick, composed of hyphae and inflated cells, hyphae 2–5 μm wide, inflated cells 14–24 × 5–9 μm. Stipitipellis gelatinous, stipe hyphae 2–7 μm wide, thin-walled, cylindrical, weakly interwoven. Clamp connections present in all tissues.

##### Habitat, ecology and distribution.

Solitary to gregarious on soil in coniferous and broad-leaved mixed forest or evergreen broad-leaved forest, known from Hunan and Hubei, China; August.

##### Additional specimens examined.

China, Hunan Province: Yongshun County, Xiaoxi National Nature Reserve, at 28.4215–28.5355°N, 110.650–110.2135°E, alt. 1000–1300 m, 30 August 2014, P. Zhang, (MHHNU 8349); Hubei Province: Hefeng County, Xiaping Town, at 30.046382°N, 110.136712°E, alt. 1223 m, 2 August 2020, Z.H. Chen, P. Long and S.N. Li, (MHHNU 32148).

##### Notes.

*Cortinariuspseudosalor* is easily misidentified as *C.salor* for their high morphological similarity, except the former has smaller coarsely verrucose basidiospores. Besides, *C.pseudosalor* distributed in Central China under coniferous and broad-leaved mixed forest or evergreen broad-leaved forest at alt. 1000–1413 m.

#### ﻿ Cortinarius subtropicus

Taxon classificationFungiAgaricalesCortinariaceae

P. Long & Z.H. Chen sp. nov.

D523DD7B-2882-534F-A4D3-5E6ACA35006B

 850394

Figs 6 , 7

##### Etymology.

*Subtropicus* (Latin) refers to subtropical distribution range of the species.

##### Holotype.

China, Hunan Province: Sangzhi County, Badagongshan National Nature Reserve, at 29.050057°N, 110.477119°E, alt. 1642 m, 29 July 2022, Z.H. Chen, J. Wen and Z.J. Jiang, (MHHNU 33533).

**Figure 6.**
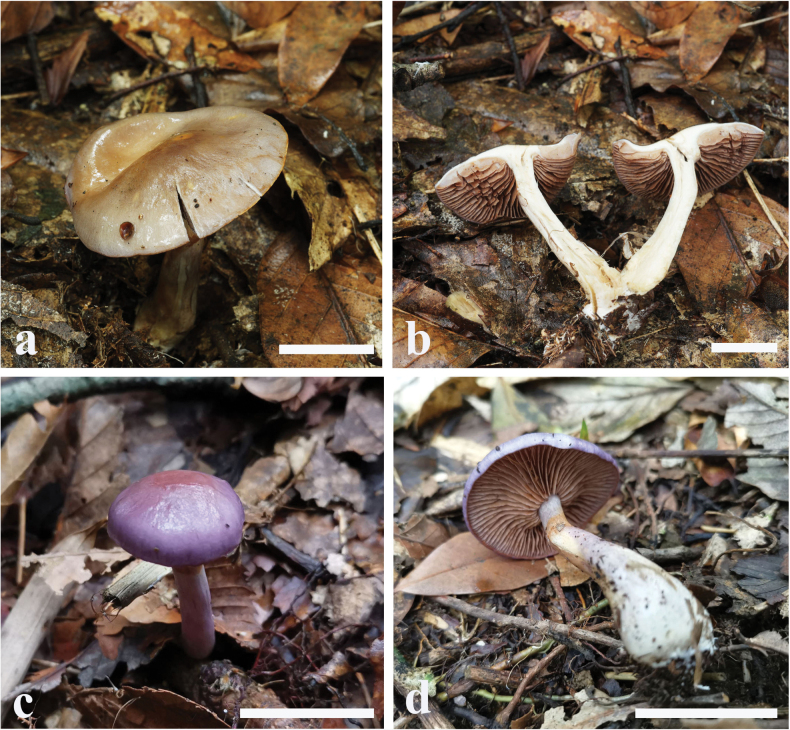
Basidiomes of *Cortinariussubtropicus* (**a, b**MHHNU 33533 **c, d**MHHNU 31964).

##### Diagnosis.

Differs from the other species of sect. Delibuti species in having an epicutis pileipellis that can be easily separated from the context of the pileus.

**Figure 7.**
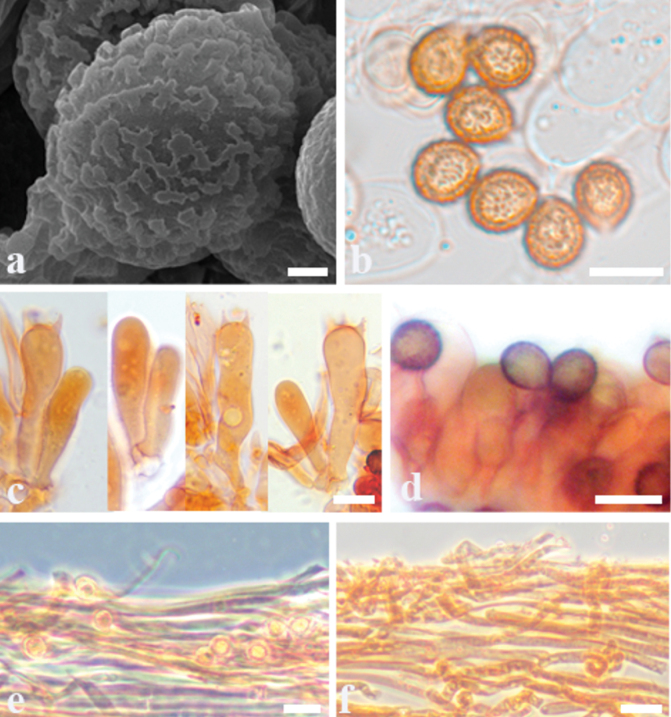
Microscopic features of *Cortinariussubtropicus* (MHHNU 33533) **a** scanning electron micrograph of basidiospores **b** basidiospores **c** basidia with probasidium **d** lamellae edge **e** stipitipellis **f** pileipellis. Scale bars: 1 μm (**a**); 10 μm (**b–e**); 20 μm (**f**).

##### Description.

Basidiomes small, development type stipiocarpic. Pileus 2.1–4.6 cm, at first broadly convex, then lower convex to plane, broadly umbonate at the centre, margin incurved; at first violaceous (15A4–15B7), tinged brown (6A5–6C7) at the centre then becoming orange brown (5B2–5B6), brown (5A4–5B6) universal veil remains at margin; surface smooth when dry or glutinous when wet, pileipellis is easy to separate. Context thin, creamy white, soft, beige (3A1–A2) when bruised. Lamellae adnate, bluish violet (18A2–18B2) with pale greyish (18B1) to brownish (5A4–5B7), rust brown (5C7) when dry, moderately distant. Stipe cylindrical to weakly clavate, bend, gradually slender to the apex, 6.4–7.2 cm long, 0.5–1.0 cm wide, lilac (15A2–15B2) when young, dirty white at maturity, leaving an ochraceous (5D7) to orange (5B6) ring on the upper stem, hollow in centre, crumbly. Odour indistinct.

Basidiospores [120/6/6] (6.6–) 7.0–9.1 (–10.3) × (5.9–)6.1–7.9 (– 10.3) μm, av. 7.8 × 6.4 μm, Q = 1.10–1.38 (1.41), Qm = 1.24 ± 0.01, subglobose to ellipsoid, yellowish brown, moderately verrucose, without amyloid and dextrinoid reaction. Basidia 32–48 × 9–12 μm, 4-spored, sterigmata 2.8–4.9 μm, clavate to subcylindrical, colourless or with amber yellow oily inclusions. Pileipellis duplex, hyphae 4–8 μm wide, epicutis gelatinous, 30–40 μm thick, composed of colourless or amber yellow, irregularly arranged and moderately interwoven hyphae, hypocuits 130–200 μm thick, composed of colourless or amber yellow, nearly parallel cylindrical hyphae. Lamellar edges fertile. Cystidia absent. Lamellar trama regular, 40–80 μm thick, composed of parallel arranged hyphae, hyphae 3–6 μm wide. Stipitipellis gelatinous, stipe hyphae 3–6 μm wide, thin-walled, cylindrical, subparallel arranged. Clamp connections present in all tissues of the basidiome.

##### Habitat, ecology and distribution.

Solitary to gregarious on soil in under evergreen broad-leaved forest, on the ground, known from Hunan, China; July.

##### Additional specimens examined.

China, Hunan Province: Sangzhi County, Badagongshan National Nature Reserve, at 29.683144°N, 109.754104°E, alt. 1645 m, 27 July 2020, Z.H. Chen, P. Long and S.N. Li, (MHHNU 31954). China, Hunan Province: Sangzhi County, Badagongshan National Nature Reserve, at 29.6767°N, 109.750696°E, alt. 1625 m, 28 July 2020, Z.H. Chen, P. Long and S.N. Li, (MHHNU 31964). China, Hunan Province: Sangzhi County, Badagongshan National Nature Reserve, at 29.676642°N, 109.750674°E, alt. 1625 m, 28 July 2020, Z.H. Chen, P. Long and S.N. Li, (MHHNU 31981). China, Guangxi Province: Xingan County, Maoershan National Nature Reserve, alt. 1900 m, 24 July 2012, X.B. Liu, (KUN-HKAS 75760).

##### Notes.

*Cortinariussubtropicus* has an epicutis pileipellis that can be easily separated from the context of the pileus. Besides, pileus become brown without lilac or dark olive tint at maturity comparing with other members of section Delibuti.

### ﻿A key to species of Cortinariussect.Delibuti

**Table:** 

1	Only distributed in the Northern Hemisphere	**2**
–	Only distributed in the Southern Hemisphere or distributed both in Northern and Southern Hemisphere	**12**
2	Pileus usually ochraceous yellow without bluish hue	**3**
–	Pileus usually bluish violet, sometimes yellow brown	**4**
3	Lamellae usually bluish violet at first, veil yellow to pale brown	***C.delibutus***
–	Lamellae pale ochraceous with tinge of pinkish violet, veil not yellowish	***C.illibatus***
4	Distributed in Europe ± North America	**5**
–	Distributed in China, East Asia	**8**
5	Growing under coniferous trees and broadleaved trees; pileus bluish violet	**6**
–	Only growing under coniferous trees (*Abies* and *Picea*); pileus usually orange	***C.largodelibutus***
6	Basidiomes small to medium-sized, pileus deep bluish violet to ochraceous; Veil violet to ochraceous	**7**
–	Basidiomes small, pileus yellow to olive-ochre at the centre, greyish blue to violet at margin then fading quickly; veil yellow	***C.betulinus***
7	Pileus usually olive brown; growing under coniferous trees (*Picea*) and broadleaved trees (*Betula*)	***C.transiens***
–	Pileus usually deep bluish violet; growing under broadleaved trees (*Quercus*, *Fagus*, *Corylus*)	***C.salor***
8	Pileus usually dark olivaceous to brown at maturity; distributed in Tibetan Plateau of China	***C.tibeticisalor***
–	Pileus usually ochraceous yellow at maturity; distributed in Central China ± Eastern China	**9**
9	Pileus with fibrils, basidiospores broadly globose to long ellipsoid, rarely subglobose	***C.fibrillososalor***
–	Pileus without fibrils, basidiospores subglobose to broadly ellipsoid	**10**
10	Basidiospores average length >8 μm	***C.subsalor***
–	Basidiospores average length <8 μm	**11**
11	Pileipellis is easy to separate; epicutis of pileipellis less than 40 μm thick, distributed from 1625 m to 1900 m	***C.subtropicus***
–	Epicutis of pileipellis more than 50 μm thick, distributed from 1000 m to 1413 m	***C.pseudosalor***
12	Growing under trees of *Nothofagus*	**13**
–	Growing under trees of Myrtaceae	**15**
13	Pileus glutinous, greyish yellow to greyish orange, stipe violet, then becoming white to pale brownish, basidiospores ellipsoid, distributed in Northern and Southern Hemisphere	***C.illitus***
–	Pileus viscid, with a green hue; basidiospores subglobose, distributed in Southern Hemisphere	**14**
14	Pileus blue‒green to aerugineous; stipe blue green; distributed in Australasia	***C.tessiae***
–	Pileus dark green; stipe white with a purple hue; distributed in New Zealand	***C.viridipileatus***
15	Basidiomes distinctly viscid to glutinous, stipe viscid, mainly greyish blue-green	***C.rotundisporus***
–	Basidiomes weakly viscid, stipe often dry, mainly yellow-green to olive	***C.calaisopus***

## ﻿Discussion

In this study, three species of Cortinariussect.Delibuti, namely *C.fibrillososalor*, *C.pseudosalor*, and *C.subtropicus*, were described as new to science based on phylogenetic analyses and morphological characteristics. The phylogenetic relationships of *C.fibrillososalor* and *C.subtropicus* in C.sect.Delibuti were resolved with close phylogenetic relationship with *C.tibeticisalor*. However, the phylogenetic position of *C.pseudosalor* is still unclear as no supported sister relationship was revealed in the phylogenetic analysis.

*Cortinariusfibrillososalor*, *C.pseudosalor*, *C.salor*, *C.subsalor*, *C.subtropicus* and *C.tibeticisalor* have morphological homogeneity of the basidiomes. The macromorphological characters of *C.pseudosalor*, and *C.salor* are similar to basidiomes, coloured bluish violet, while *C.subsalor* is coloured purple to purplish red in pileus centre. Besides, *C.pseudosalor* has smaller coarsely verrucose basidiospores comparing *C.salor* and *C.subsalor* (Kibby and Tortelli 2021; Xie et al. 2021a). Meanwhile, *C.salor* is characterized by its lilaceous lamellae all the time and the narrow distribution in European woodlands, while *C.pseudosalor* and *C.subsalor* occurs in Asia (Xie et al. 2021a). *Cortinariusfibrillososalor* with violaceous to whitish mauve tint differ from other species in this section in the appearance of fibrils on the pileus and its broadly globose to long ellipsoid basidiospores (Kibby and Tortelli 2021; Xie et al. 2021a). *Cortinariussubtropicus* was found in the subtropical monsoon climate region of the Hunan and Guangxi provinces distributed from 1625 m to 1900 m. *Cortinariustibeticisalor* was only distributed in Tibetan Plateau and was usually olivaceous to brown at maturity, while olivaceous species in sect. Delibuti mainly occurred in the South Pacific (Soop et al. 2019; Xie et al. 2021a).

Phylogenetic analysis was first applied to the taxonomic study of *Cortinarius* with ITS (Liu et al. 1997). Later, phylogenetic relationships were inferred mainly based on ITS, LSU sequences, and *rpb1*, *rpb2* were also confirmed to help elucidate the relationships of species in *Cortinarius* (Frøslev et al. 2005; Soop et al. 2019; Xie et al. 2022). Species delimitation could be justified by the combination of ITS and LSU sequences (Nilsen et al. 2021; Zhou et al. 2023), a two-locus dataset (ITS and LSU) was used for the research of three new species and their similar species in the present study. However, it needs more sequence data and DNA markers for recognising higher taxonomic rank such as subgenus or genus. In section rank, a two-locus dataset (ITS, LSU) and four-locus dataset (ITS, LSU, *rpb1* and *rpb2*) were first employed for phylogenetic analyses, and the latter provided a higher BP value and clearer tree structure, although with limited *rpb1* and *rpb2* (Soop et al. 2019). Besides, the first phylogenomic study based on shallow whole genome sequencing was conducted for Cortinariaceae revision (Liimatainen et al. 2022).

## Supplementary Material

XML Treatment for Cortinarius fibrillososalor

XML Treatment for Cortinarius pseudosalor

XML Treatment for Cortinarius subtropicus

## ﻿Additional information

### Data availability

All of the data that support the findings of this study are available in the main text or Supplementary Information. The sequence data generated in this study are deposited in NCBI GenBank.

## Supplementary materials

Supplementary material 1 Multiple sequence alignment Data type fas https://binary.pensoft.net/file/968973This dataset is made available under the Open Database License (http://opendatacommons.org/licenses/odbl/1.0/). The Open Database License (ODbL) is a license agreement intended to allow users to freely share, modify, and use this Dataset while maintaining this same freedom for others, provided that the original source and author(s) are credited.Pan Long, Song-Yan Zhou, Sai-Nan Li, Fei-Fei Liu, Zuo-Hong Chen
